# Lessons Learned from the COVID-19 Pandemic and How Blood Operators Can Prepare for the Next Pandemic

**DOI:** 10.3390/v14102126

**Published:** 2022-09-27

**Authors:** Steven J. Drews, Sheila F. O’Brien

**Affiliations:** 1 Canadian Blood Services, Microbiology, Donation and Policy Studies, Canadian Blood Services, Edmonton, AB T6G 2R8, Canada; 2 Division of Applied and Diagnostic Microbiology, Department of Laboratory Medicine and Pathology, University of Alberta, Edmonton, AB T6G 2R3, Canada; 3 Epidemiology and Surveillance, Donation Policy and Studies, Canadian Blood Services, Ottawa, ON K1G 4J5, Canada; 4 School of Epidemiology and Public Health, University of Ottawa, Ottawa, ON K1N 6N5, Canada

**Keywords:** blood operators, blood-borne agents, pandemic planning, emerging viral pathogens

## Abstract

Humans interact with virus-infected animal hosts, travel globally, and maintain social networks that allow for novel viruses to emerge and develop pandemic potential. There are key lessons-learned from the coronavirus diseases 2019 (COVID-19) pandemic that blood operators can apply to the next pandemic. Warning signals to the COVID-19 pandemic included outbreaks of Severe acute respiratory syndrome-related coronavirus-1 (SARS-CoV-1) and Middle East respiratory syndrome-related coronavirus (MERS-CoV) in the prior two decades. It will be critical to quickly determine whether there is a risk of blood-borne transmission of a new pandemic virus. Prior to the next pandemic blood operators should be prepared for changes in activities, policies, and procedures at all levels of the organization. Blood operators can utilize “Plan-Do-Study-Act” cycles spanning from: vigilance for emerging viruses, surveillance activities and studies, operational continuity, donor engagement and trust, and laboratory testing if required. Occupational health and donor safety issues will be key areas of focus even if the next pandemic virus is not transfusion transmitted. Blood operators may also be requested to engage in new activities such as the development of therapeutics or supporting public health surveillance activities. Activities such as scenario development, tabletop exercises, and drills will allow blood operators to prepare for the unknowns of the next pandemic.

## 1. Introduction

The COVID-19 pandemic originated in late 2019 as the result of the emergence and transmission of the Severe acute respiratory syndrome-related coronavirus-2 (SARS-CoV-2) virus from an animal host to humans [1,2]. This emergence event was not unexpected as in the past two decades health systems had previously struggled with Severe acute respiratory syndrome-related coronavirus-1 (SARS-CoV-1) in 2003 [3], influenza A H1N1 (pdm09) in 2009–2010 [4,5], and Middle East respiratory syndrome-related coronavirus (MERS-CoV) in the 2010′s [6,7]. None of these agents were primarily blood-borne and none were capable of being transmitted by the transfusion of blood products [8,9]. The COVID-19 pandemic has impacted on all elements of society. Blood operators have played an important role in studying and characterizing the COVID-19 pandemic and can provide lessons learned from this experience [10,11,12,13,14]. 

Humans knowingly and unknowingly interact with or encroach on virus-infected animal hosts [15]. We also have improved access to global travel options. Globalization of travel and complex human social networks provide the opportunity for infected individuals to spread emerging viruses broadly and quickly [16,17,18,19]. Blood operators must seek to avoid a scenario where the emergence of a viral pathogen goes undetected for a significant period (e.g., months to years) and leads to significant transfusion transmission. For example, during the early days (early 1980s) of the human immunodeficiency virus (HIV) pandemic, the etiology of acquired immunodeficiency syndrome (AIDS) was unknown and seemingly well donors were transmitting the virus [20]. In the era before molecular medicine, it would take over two years (1985) to develop an approved test once the medical community became aware of AIDS [21]. In the United States (US), the first nucleic acid tests for HIV were implemented years later in 1999 [22]. There were technological and knowledge gaps in understanding HIV and blood product transfusion led to large numbers of transfusion transmissions. By 1984, hemophilia patients in the US were estimated to have high incidence rates (3.6 cases/1000 hemophilia A patients and 0.6 cases/1000 hemophilia B patients) [21]. Four decades later combined serology and nucleic acid (NAT) testing and donor deferrals in Western countries have resulted in extremely low levels (e.g., 1 per 21.4 million donations in Canada, 2012–2014 estimate) [23].

This manuscript will focus on the lessons learned from the COVID-19 pandemic and provide suggestions for how blood operators can prepare for the emergence of novel viruses that may cause the next viral pandemic.

## 2. Lessons Learned

### 2.1. Emerging Zoonotic Viruses Should Be Routinely Tracked by Blood Operators

In this manuscript we will define an emerging pathogen “as an infectious agent whose incidence is increasing following its first introduction into a new host population.” In contrast, a “re-emerging pathogen is one whose incidence is increasing in an existing host population as a result of long-term changes in its underlying epidemiology” [24]. A prior analysis suggested that the majority (75%) of emerging pathogens were zoonotic and zoonotic agents were twice as likely to be emerging pathogens as non-zoonotic agents [25]. In the last 50 years, apart from SARS-CoV-2, health systems have been impacted by a variety of emerging and re-emerging zoonotic viruses. Both HIV-1 and HIV-2 emerged in humans with an ensuing HIV-1 pandemic following multiple cross-species transmissions of simian immunodeficiency viruses (SIVs) that naturally infected African primates [26]. There is also now a strong consensus that the emergence of the closely related SARS-CoV-1 in 2002–2003 was also due to a human-animal interaction [27]. The re-emergence of filoviruses such as Ebola virus appear to have a complex network of animal hosts including non-human primates [28]. Influenza A H1N1 (pdm09) emerged following a quadruple reassortment involving human, avian and swine-origin viruses [29]. Around the time of emergence of SARS-CoV-2 in the human population, there was wide diversity of circulating alphacoronaviruses and betacoronaviruses described in animals [30]. A variety of small mammals may act as hosts for Monkeypox virus and enable emergence in new settings in Central and West Africa [16].

Considering these trends, during the period prior to the emergence of SARS-CoV-2, Canadian Blood Services maintained a spreadsheet of emerging pathogen events and routinely scanned the peer-reviewed and grey literature for patterns of zoonotic disease emergence or re-emergence (Table 1). In the Winter of 2019–2020 we reached out to public health colleagues and virologists and used the grey and peer-reviewed to understand more about an odd cluster of respiratory diseases in China. These respiratory diseases cluster would later become the COVID-19 pandemic and would be attributed to SARS-CoV-2. Although controversies exist on the animal species and events that occurred to create this emergence, it is clear that this virus emerged from a zoonotic source [1,31,32] (Table 1).

### 2.2. Understand How the Emerging Virus of Interest Is Transmitted in the Community

Emerging and re-emerging pathogens are a risk to society because their pathophysiology may not be understood [50], individual and population immunity may not exist [51], public health organizations may have no template of remediation activities to draw from [52], there may be no appropriate or pathogen specific treatment that exists [53], means of transmission and appropriate infection control activities may need knowledge and time to develop [54], and diagnostic tools and laboratory tests (serology [55] and/or nucleic acid tests [NAT] [56]) may not exist or be limited. Canadian blood services utilized taxonomic information as well as early epidemiologic information to understand that SARS-CoV-2 was primarily transmitted via the respiratory route [32,33] (Table 1).

### 2.3. Determining Whether the Emerging Virus Is a Direct Threat to the Blood Supply

Early in 2020, there was a significant amount of work undertaken to determine whether there was a risk of blood-borne transmission of SARS-CoV-2. The blood banking community concluded that it was unlikely that SARS-CoV-2 was transfusion-transmissible [9,42,57,58]. (Table 1).

The next pandemic will require an early understanding of (1) whether the virus has a viremic phase in humans [41,42]; (2) whether blood specimens contain viable virus (via animal or tissue culture experiments) [41,58] and (3) whether there is evidence for transfusion-transmission following post-donation information from donors who donated while symptomatic [41]. If the next pandemic virus is indeed transfusion-transmissible, then some jurisdictions will have surveillance data to undertake risk analyses. Depending on the natural transmission dynamics (e.g., is the agent transmitted by vector bite?), other blood operators may need to undertake their own surveillance in their own blood donor populations using NAT and/or serology [48]. These risk calculations can then be used in an Alliance of Blood Operators risk-based decision-making process to determine the best risk-mitigation steps to proceed with in terms of donor screening questions, donor deferrals, and donor testing for the virus of concern [59].

### 2.4. Be Vigilant for Viral Genetic Changes in Viruses That Might Impact on Blood Operator Practices

Viruses can be most easily described as delivery systems for nucleic acid coded information that is under constant evolutionary pressure to change or mutate [60]. Mutation rates associated with viral evolution can be broadly associated with the composition of the viral genome (Table 1). Viral mutation rates help drive the transmission of viruses and evade human and animal host responses [61,62]. In general, mutation rates highest in single stranded (ss) ribonucleic acid (RNA) viruses (e.g., SARS-CoV-2) when compared to double stranded (ds) RNA, ss deoxyribonucleic acid (DNA) and ds DNA viruses. There is also considerable rate variation among RNA viruses when substitution rates are characterized as substitution per site per year [62,63]. As with SARS-CoV-2, there may be multiple pressures driving the evolution of an emergent virus (e.g., human immunity) and it may be difficult to predict the trajectory of a virus from emergence to intra-pandemic periods [1,61,64].

Changes in the dominant SARS-CoV-2 variants of concern over time meant that blood operators needed to review policies focussed on enhancing staff safety and reducing transmission in the workplace [36,37] (Table 1). We also spent considerable time understanding the safety of vaccine and the importance of vaccination in reducing the burden of disease in society [38,39,40]. Continual reviews of the literature by subject matter experts allowed for the development of policies and processes to ensure a safe environment for staff and donors. Finally, we also tracked the literature to ensure that genetic or strain changes in SARS-CoV-2 did not change the transfusion-transmission risk profile [41,42].

### 2.5. Blood Operators May Be Involved in Creating New Blood Products

Even if the next pandemic is not blood-borne and/or transfusion-transmitted blood operators may face business pressures that will require agility and creativity. Early in the COVID-19 pandemic, there were no COVID-19 vaccines nor COVID-19 specific antiviral agents available to reduce severe outcomes in infected individuals (Table 1). This led to some researchers to initiate clinical trials to assess whether COVID-19 convalescent plasma (CCP) could be an effective therapy for patients hospitalized for SARS-CoV-2 infections [43,44]. A survey of International Society of Blood Transfusion (ISBT) members published in 2022 noted that 52 institutions collected CCP [45]. As a result, blood operators needed to identify and develop approaches to qualify blood donors for CCP donations when no gold standard COVID-19 serological tests existed [46,47]. This included creating deferral policies for CCP donors who were convalescing after SARS-CoV-2 infection [65]. Blood operators developed early expertise in characterizing SARS-CoV-2 serological assays [10,51,55]. In Canada, this early expertise led to blood operators becoming involved with large SARS-CoV-2 seroprevalence studies and COVID-19 focussed research activities [48] that still continue into late 2022 [10,12,66]. Understanding the Further development of the role blood operators play in supporting public health is important both to maximise the value of blood services, but also to ensure that blood operators are at the table as national and international pandemic plans are developed and reviewed.

### 2.6. Blood Operators May Be Asked to Play a Public Health Role

Early in the pandemic, Canadian Blood Services became engaged with public health surveillance activities focussed on generating SARS-CoV-2 seroprevalence data and undertaking an analysis of humoral correlates of immunity (Table 1). These activities utilized residual donor specimens that were tested in a research setting outside of the regulated blood operator testing environment [10,11,48,51,66,67]. In the next pandemic, blood donor specimens may provide a unique perspective on the seropositivity of healthy individuals on a scale across more that one public health region. Blood operators should be evaluating how representative their donor populations are of the general population and planning ways to enhance data collection [48,49].

## 3. How Blood Operators Can Prepare for the Next Pandemic

### 3.1. Be Prepared for Changes in Activities, Policies, and Procedures

Practically, this means that blood operators need to be prepared for changes in activities, policies, and procedures during a pandemic.

### 3.2. Activities

Viral evolution could theoretically change the pathophysiology of the virus, and this may change the potential for transfusion transmission and blood operators may need to decide if and when they should use laboratory tests to test blood products [9].

### 3.3. Policies

Blood operator policies focussed on staff vaccination requirements will need to stay current to viral evolution that changes the effectiveness of vaccines when available [68].

### 3.4. Procedures

Although SARS-CoV-2 was not transfusion-transmitted, there was an initial concern whether clinical tests would be consistently able to detect the virus [69,70]. If the next pandemic virus is transfusion-transmitted, and laboratory NAT is required for the release of blood products, blood operators will need to ensure that their assays keep pace with viral evolution.

### 3.5. Models for Pandemic Planning Improvement

**Figure 1:**
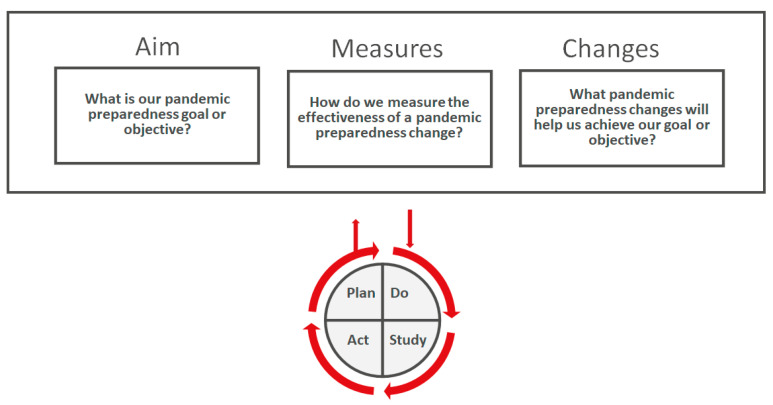
Four steps in a pandemic planning MFI. Pandemic planning is not a static process and required clear aims with well-designed proposed changes in practice. Effective pandemic planning also requires an understanding of how to measure implemented changes. PDSA cycles allow for incremental tests of change.

The Agency for Healthcare Research and Quality Identifies a “Model for Improvement” (MFI) that can be used to generate a framework to organize improvement work. For pandemic planning, four MFI steps can be generated; (1) What is our pandemic preparedness goal or objective? to the Model for Improvement, (2) Determine how to measure the effectiveness of a pandemic preparedness change, (3) Determine the pandemic preparedness changes that will help us achieve our goal or objective, (4) Undertake small tests of using Plan-Do-Study-Act (PDSA) cycles (Figure 1) [71].

### 3.6. Approaches to Protect the Blood Supply

**Figure 2:**
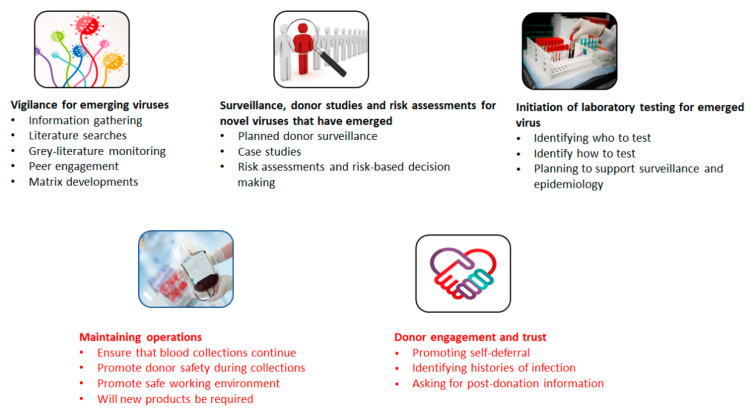
Activities that support pandemic preparedness. To ensure the safe supply of blood products, blood operators undertake complex activities on a routine basis. Pathogen-specific areas of focus (black text) include vigilance for emerging viruses, surveillance, donor studies and risk assessments for viruses that have emerged and initiating laboratory testing for emerging viruses. Core activities to ensure the adequate supply of blood products (red text) include donor engagement and trust, and maintenance of operations. Individuals working in blood operator settings may be engaged in one or more of these activity areas. Resources allotted to areas of focus may depend on a variety of factors including whether the emerging pathogen is transfusion-transmitted, or the level of disease spread in a donor population or work force.

During the development of a pandemic, blood operators need to take a structured approach to planning for all stages from emergence of a novel virus to an active pandemic period. Figure 2 identifies processes that blood operators can utilize in “Plan-Do-Study-Act” cycles spanning from:

- vigilance for emerging viruses
- surveillance, donor studies and risk assessments for novel viruses that have emerged
- ensuring that operations continue
- donor engagement and trust
- initiation of laboratory testing for emerged virus

Blood operators must face these issues when undertaking all facets of operations. In addition to these concerns, blood operators may be faced with a question as to whether the emerging or re-emerging agent is blood-borne and capable of being transmitted by the transfusion of blood products [9,57]. A variety of variables may also create conditions that reduce the blood supply to support the healthcare system in general [72,73].

### 3.7. Hardwiring Vigilance by Creating or Updating a Pandemic Plan?

Prior to the COVID-19 pandemic, there were a variety of influenza pandemic plans focussed on the health services sector that were developed at national and international levels [74,75,76]. Early in the influenza A H1H1(pdm09) there were calls by risk management professionals for organizations to “dust off” their pandemic business continuity plans [77]. This continued throughout seasonal influenza outbreaks [78]. Early in 2020, there were once again calls for organizations to “refresh” their pandemic plans [79]. In Canada, Canadian Blood Services was quickly able to review its pandemic influenza plan for the organization, adjust the plan for COVID-19 and present the plan to its leadership team for a review. The primary set of adjustments moved away from an influenza wave approach where epidemiologic triggers guided different approaches by the business. The new plan noted that triggers for changes on business activities would have to rely on more organic decision making because in early 2020 the epidemiology of SARS-CoV-2 was not well understood.

### 3.8. Plan for the Long Term: The End May Not Be Near

The SARS-CoV-2 pandemic as well as the prior Influenza a H1N1 (pdm09) pandemic presented with multiple waves of activity [80,81]. Generally, pandemic plans have approaches for (1) early pandemic periods (virus has recently emerged with no immunity in population), (2) mid-pandemic periods (multiple waves of the pandemic have occurred as virus evolves, growing immunity in population), (3) late pandemic periods (viral evolution is balances by a high level of immunity in population) and (4) post-pandemic periods (virus evolution is countered by host immunity, virus may become endemic or disappear) [74,82]. Pandemic planning for blood services should include processes and touch points for interaction with public health authorities, and ideally be co-developed with public health plans. Pandemic plans developed early after the emergence and establishment of a pandemic virus may not have an easily identifiable endpoint. Virus fitness may change dramatically over the course of a pandemic [83] and emerging lineages may emerge even in the presence of high levels of population vaccination [84]. This means that the plan may identify actions for early and middle periods of a pandemic but may not inform organizations on how to de-escalate from a pandemic. Planners may need to emphasize that they may not be able to identify when pandemic restrictions can be removed. Practically this impacts on return-to-work strategies for employees [85,86,87,88,89], mandatory vaccination strategies [90], approaches to masking policies [91], and organization infection control strategies [92]. As viruses evolve during a pandemic, it may be necessary to re-assess risk calculations and approaches to protect blood safety.

### 3.9. Plan Broadly Even if the Next Pandemic Is Not Blood-Borne and/or Transfusion-Transmitted

Depending on the mode of transmission of the next pandemic virus, blood operators will need to undertake activities, policies, and procedures to ensure the safety of staff and donors at donations sites as well as operational facilities. Blood donor deferrals were created not only as an extremely precautionary approach to prevent transfusion-transmission but also to prevent respiratory transmission of SARS-CoV-2 in donor centers [93]. A survey of ISBT members noted that measures to protect blood donors and staff included monitoring donors for signs and symptoms of COVID-19, minimizing consumption of fluids and salty snacks on sites and increasing frequencies of surface cleaning [45]. Donor deferral policies based on exposure risk, COVID-19 disease and travel were also introduced by blood operators [93]. Decreases in blood donations prompted blood operators to consider strategies and approaches to improve donor recruitment [45,94,95]. The introduction of COVID-19 vaccines required blood operators to review the characteristics of each vaccine and determine if donors should be deferred after vaccine as well as the duration of any deferral periods [96]. To ensure a safe work environment, blood operators also struggled with vaccination policies for staff at all levels of the organization.

### 3.10. Consider Candidate Agents for the Next Pandemic

A variety of pathogens may be candidate agents of the next pandemic. Although a novel agent may emerge there are several existing candidates that may cause the next pandemic. This could be due to changes in fitness, transmission dynamics or infection control failures. Existing agents could include Ebola virus or an avian influenza A virus strain [97,98]. Viruses that can be transmitted by a respiratory route are good candidates because of the ease of transmission of these viruses via respiratory droplets and aerosols. Leading candidates might involve a zoonotic introduction followed by mutations that enhanced human infections and ensure improve viral fitness [83,99]. It may even be possible that next pandemic may also be caused by a zoonotic betacoronavirus [98].

Severe acute respiratory syndrome-related coronavirus-2 (SARS-CoV-2) is a single stranded positive sense RNA virus within the Realm *Riboviria*, Kingdom *Orthornaviria*, Order *Nidovirales*, Family *Coronaviridae*, Genus *Betacoronavirus*, Subgenus *Sarbecovirus*, Species *Severe acute respiratory syndrome-related coronavirus*. It shares the same species with Severe acute respiratory syndrome-related coronavirus-1 (SARS-CoV-1) [33]. Within the same genus, in the Subgenus *Merbecovirus* sits the species *Middle East respiratory syndrome-related coronavirus* (MERS-CoV). Nearby in the same genus within the Subgenus *Embecovirus* sits the seasonally circulating *Human coronavirus HKU1* [33,100]. There are also three other genera within the Family *Coronaviridae* (*Alphacoronavirus*, *Gammcoronavirus*, *Deltacoronavirus*), the last major coronavirus emerging events or crises have involved viruses within the genus *Betacoronavirus*. Coronaviruses have large genomes that exhibit a high level of plasticity and are prone to recombination. This makes them prone to spread between animal hosts [101].

The pathophysiology of coronavirus even within a genus may vary and newly discovered betacoronaviruses that emerged around the time of SARS-CoV-2 may not bind to angiotensin-converting enzyme 2 (ACE-2) due to genetic deletion in two regions of their receptor-binding motifs [30]. With changing host cell receptor binding profiles and rapidly developing mutations to enhance viral fitness like its cousin SAR-CoV-2 [83], a new emerging betacoronavirus might still be transmitted via a respiratory route but may present with different signs and symptoms than SARS-CoV-1, MERS-CoV or SARS-CoV-2. At this point in time, a discussion on whether a newly emerged pandemic betacoronavirus might be transmitted by other routes (gastrointestinal, transfusion transmission) is speculative.

Both the COVID-19 pandemic and the recent spillover of Monkeypox virus, an Orthopox virus, into non-endemic countries in 2022 raises questions about how blood operators can remain vigilant and prepared for the next emerging virus threat that could lead to a pandemic [102]. Currently, we have no evidence that either SARS-CoV-2 or Monkeypox are transfusion transmitted [8,9]. Based on experimental data we also know that SARS-CoV-2 as well as Orthopox viruses may be pathogen reduced in non-red blood components using existing pathogen reduction methods [103,104,105]. It is unknown whether Monkeypox virus will drive a global pandemic [106].

## 4. What to Watch for in a Worst-Case Scenario

Planning for worst case scenarios will allow for blood operators to think about warning signals suggesting transfusion transmission of a new emerging virus with pandemic potential. There are multiple factors that could impact on whether an emerging betacoronavirus might be transfusion transmitted. They could include factors such as; reproductive number, transmissibility, disease severity, host cell receptor, and vaccine effectiveness [107]. One scenario of concern would be the emergence of a highly mutable emerging virus that was found in extremely high concentrations in both the upper and lower respiratory tract. This virus might also be primarily aerosol transmitted would be more likely to cause lower respiratory tract infections [108]. Large numbers of individuals could be then infected even if they maintained effective social distancing and wore surgical or procedure masks. A resulting high reproductive number (Ro) might allow for new variants to quickly emerge thereby complicating attempts to developing a broadly protective vaccine. An asymptomatic infectious period may allow individuals to spread the virus unknowingly [109]. Once infections occurred, an affinity to multiple host cell receptors and an aggressive infectious process might cause extensive lower respiratory tract damage [110]. The scientific community should be watching for evidence of an asymptomatic phase of pneumonia in some individuals [111]. In this case, the concern would be that extensive lung damage in individuals who felt well enough to donate blood products might lead to viable virus spilling over into the circulatory system. If the virus was then transfused to individuals, a concerning scenario would be if the virus could use a diversity of receptors to infect multiple organ systems and cause extensive systemic damage.

## 5. Conclusions

Blood operators are key members of the health care system and often play an underappreciated public health role [48]. Strengthening and further developing a mutually beneficial role with public health should be a priority. Although the SARS-CoV-2 pandemic is still ongoing (as of September 2022) there are key lessons-learned that blood operators can apply to the next pandemic. As individuals and organizations, we should be prepared for more emerging viral threats such as SARS-CoV-2 which emerged from an animal host and jumped into the human population [1,30]. When an emergence event occurs, we should remember that viruses are prone to constant evolution which can impact on disease pathophysiology, host range and epidemiology [60,109,110]. These events may quickly turn an emerging virus of concern into a virus with pandemic potential. With changing viral evolution blood operators should be prepared for changes in activities, policies, and procedures at all levels of the organization [9,68,69,70]. Planning for worst case scenarios will allow for blood operators to think about warning signals suggesting transfusion transmission of a new emerging virus with pandemic potential.

## Figures and Tables

**Table 1:** Issues, causes and lessons learned by blood operators during the COVID-19 pandemic.

Issue	Possible Cause	Blood Operator Lesson for Future Emerging Event or Pandemic	References
Rapid emergence and spread of a zoonotic agent even in presence of public health controls.	Closer interactions between humans and infected host animals. Unclear epidemiology and transmissibility. Human populations travel globally.	Continue to track zoonotic virus activity and risks for human emergence and spread.	[1,25,31]
Early growing evidence that SARS-CoV-2 was spread between people via the respiratory route.	Unclear understanding of ease of virus transmission.	Look for taxonomic and early epidemiologic clues for patterns of transmission.	[32,33]
Questions from transfusion community on transfusion-transmission of SARS-CoV-2.	Unclear evidence on transmissibility. Questions from transfusion community, regulators, and public.	Undertake risk assessments for transfusion transmission. Continue to review even after first assessments done.	[9,34,35]
Evidence for small scale (single nucleotide polymorphisms) and large scale (lineage replacement) changes in virus	Nature of virus genome and responses to evolutionary pressures.	Be prepared to change practices and processes focused on staff and donor safety as viruses evolve. Consider if transfusion-transmission risk changes.	[36,37,38,39,40,41,42]
Blood operators asked to help assess new blood products	New emerging agent had no known effective treatment and vaccines were not yet available.	Be prepared to be involved in clinical trials that involve the blood operator. Be prepared to be involved in the development of new donor testing approaches.	[43,44,45,46,47]
Blood operators asked to engage with public health on surveillance initiatives	Public health may focus on data from unwell populations. Public health may focus regionally or may use aggregate data across regions collected using different approaches.	Be prepared to operationalize new surveillance strategies. Be prepared to advise how blood donors can represent the general population.	[48,49]

## Data Availability

Not applicable.
